# Intravascular Lithotripsy to Facilitate Extraction of Very Old Cardiac Implantable Electronic Devices Leads

**DOI:** 10.1111/jce.70280

**Published:** 2026-02-01

**Authors:** Jamie Kowal, Travis Richardson, George H. Crossley

**Affiliations:** ^1^ Department of Cardiovascular Diseases Vanderbilt University Nashville Tennessee USA; ^2^ Vanderbilt Heart and Vascular Institute, Vanderbilt University Medical Center Nashville Tennessee USA

**Keywords:** intravascular lithotripsy, lead extraction

## Abstract

**Background:**

One of the challenges encountered when extracting transvenous leads with long dwell times is the presence of dense calcifications encasing the leads. This is the most likely reason for failure to advance a laser extraction sheath. The Shockwave intravascular lithotripsy (IVL) device is an angioplasty balloon that delivers intravascular lithotripsy and fractures calcified lesions in the vasculature, approved for use in coronary and arterial angioplasty. It has also been reported as an adjunctive tool in transvenous lead extraction.

**Objective:**

To report the Vanderbilt University Medical Center experience using Shockwave(r) Lithotripsy in the extraction of very old leads.

**Methods:**

We report procedural outcomes in this retrospective single‐center series of 24 patients in whom IVL was performed for pretreatment before lead extraction. To use the shockwave balloon, one must have venous access along the path of the leads. In some cases, there was venous access, and in others, a lower‐risk lead was extracted first to allow for the passage of the Shockwave balloon. After IVL pretreatment, leads were extracted using conventional laser and, when necessary, mechanical tools.

**Results:**

Forty‐nine total leads were extracted, with a median of 2 leads per patient and median dwell time of 16 years with a range of 4–36 years. All transvenous leads were successfully removed, and there were no significant complications. An excimer laser system (Philips, Inc.) was utilized for extraction in all cases, with a median sheath size of 14 Fr. Despite long dwell times, mechanical extraction tools were only required in 6 (12%) of the leads.

**Conclusion:**

Our experience contributes to the growing body of data supporting the use of Shockwave IVL as an adjunctive measure during extraction of calcified leads with long dwell time.

## Graphical abstract

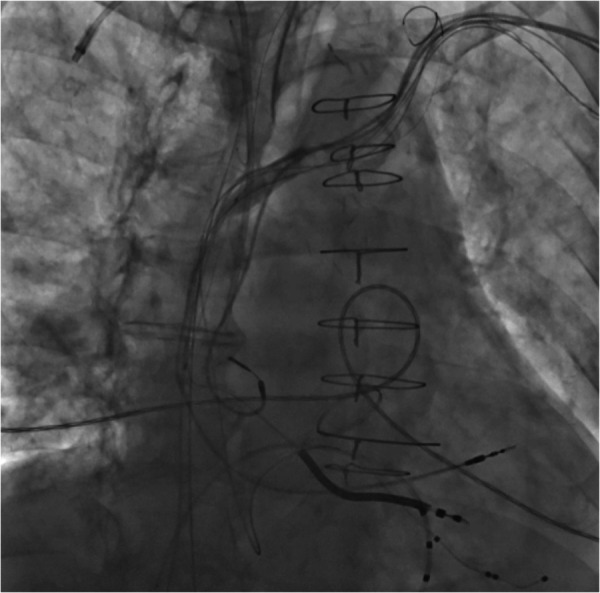

## 1 Background

The rise in implantation of cardiac implantable electronic devices (CIED) in concert with improved patient survival due to guideline‐directed medical therapy has resulted in an increased number of chronically indwelling CIED leads. With this increased lead burden comes an increase in extraction volume. Despite this, transvenous lead extraction (TLE) remains an underused technology for pacemaker and defibrillator lead management [1, 2]. While techniques and tools, including excimer laser and mechanical rotating sheaths, have improved the safety and clinical success, procedural failure and mortality rates are not negligible [3, 4].

Among the challenges encountered when extracting transvenous leads is the presence of dense calcified areas of fibrosis [5]. Long lead dwell time is associated with the formation of fibrosis encapsulating intravascular leads, which eventually leads to calcification. In addition to long dwell time, calcification is more commonly observed in younger patients and those with advanced renal disease [6, 7, 8]. Calcified fibrosis often requires increased traction forces, larger extraction tools, and the use of mechanical dissection tools. The concern with more aggressive extraction techniques is the potential for vascular injury. Even in the absence of catastrophic patient complications, microscopic examination of explanted leads has revealed evidence of transmural vein segments [9]. In addition to lead‐vessel fibrosis, lead‐to‐lead adherence is critical and can make extractions more difficult in patients with multiple indwelling leads [10]. These factors have a substantial impact on the degree of difficulty of the extraction procedure and may require crossover of one sheath technology to another tool [11, 12]. Additionally, while procedural complication rates may be low, outcomes from such complications can be catastrophic [4, 13]. Thus, technologies with the potential to ameliorate some of the challenges associated with TLE are attractive.

The Shockwave intravascular lithotripsy (IVL) system works by creating an acoustic pressure wave that radiates from the delivery catheter within an effective radius of approximately 3 to 7 mm. The resulting series of concussive waves generates a peak force of about 50 ATM over 2 to 4 μs, propagating fracture of calcified lesions in the vasculature immediately adjacent to the delivery catheter [14, 15, 16].

To date, a single‐center retrospective series evaluated the safety and feasibility of Shockwave IVL in 14 cases. There were no significant differences in baseline patient or device characteristics, with investigators demonstrating a reduced median extraction time approaching 30 min in the IVL facilitated extraction group (mean lead dwell time 11.1 years) compared to the control group (mean lead dwell time 10.8 years), with similar procedural and clinical success rates [17]. There have also been cases reported in which the use of IVL allowed for successful extraction when traditional techniques failed [18]. In this study, we present our outcomes using IVL as pretreatment prior to extraction of chronically indwelling leads.

## 2 Methods

### 2.1 Study Population

This was a retrospective cohort comprised of all patients undergoing IVL before TLE at Vanderbilt University Medical Center between June 2023 and June 2025. IVL was used at the operator's discretion and typically reserved for cases with lead dwell time greater than 15 years, radiographic evidence of lead‐lead binding, dual coil implantable cardioverter‐defibrillator lead systems, and/or chronic kidney disease.

### 2.2 Extraction Procedure and IVL Pretreatment Method

All procedures were performed under general anesthesia in a hybrid surgical operating room with a cardiac surgery team on standby.

Each patient had at least two femoral venous sheaths placed, including one of at least 12‐F diameter to allow for the emergent placement of a compliant balloon over an Amplatz Super‐stiff wire positioned in the superior vena cava. When applicable, locking stylets were advanced through the lead lumen, and the insulation was secured with surgical sutures prior to IVL pretreatment in case of potential compromise of the lead lumen during IVL.

While preprocedural CT venography was used in some of our patients, we typically used venography at the time of the procedure to assess venous patency and to help size the balloon. Next, vascular access adjacent to the leads was obtained either using an ipsilateral axillary venous approach or from the femoral vein in the case of superior vascular occlusion. An exchange length 0.018” Steelcore guidewire was placed alongside the existing leads. If a guidewire would not pass adjacent to the existing lead body, then the newest lead was extracted first to create an intravascular conduit for guidewire placement. Operators used a SupraCore guidewire (Abbott) for retention of vascular access, which was later exchanged for the Steelcore guidewire. An 8‐ to 10‐mm Shockwave L6 IVL balloon was then advanced over the guidewire, sized based on the target vessel diameter. Using the distal and proximal radiopaque markers as a guide, the balloon was positioned at sequential sites along the entire lead body spanning from the superior venacave/right atrium (SVC/RA) junction to the junction of the first rib and the clavicle. At each site, the balloon was inflated to the nominal inflation level before delivering a series of 30 pulses via the IVL system. The balloon was then deflated and repositioned before repeating this method in a stepwise fashion.

Owing to the importance of maintaining proximity of the IVL balloon to the target tissue for optimal results, pretreatment was not applied within the right atrium, coronary sinus or right ventricle.

Once the pretreatment was completed, the remainder of the extraction procedure was performed. In this series, an excimer laser sheath (Philips, Inc.) was used primarily with rotational mechanical tools added, as needed.

### 2.3 Data Collection

Patients were identified using the Vanderbilt Transvenous Lead Extraction Registry (VaTLER), a retrospective observation repository. The medical records of all patients were then manually reviewed, and those with confirmed extraction utilizing Shockwave IVL pretreatment were included in this study.

The median dwell time of the leads being targeted for extraction, as well as the total lead dwell time of all intravascular leads present at the time of the procedure were calculated for each patient.

Procedural success was defined as full removal of all targeted leads without retention of any lead components. Clinical success was defined as the ability to complete the primary goal of the procedure. When the indication for procedure was infection, clinical success required complete removal of all the device system hardware. Safety events were tracked according to the 2017 Heart Rhythm Society expert consensus statement on cardiovascular implantable electronic lead management and extraction.

### 2.4 Statistical Analysis

Statistical analysis was performed using Microsoft Excel Version 16.

### 2.5 Ethical Review

This study qualified for IRB exemption by the Vanderbilt University Medical Center institutional review board.

## 3 Results

Over the course of the study period, 24 patients with a total of 49 leads (median 2 leads per patient) underwent IVL adjuvant therapy prior to conventional lead extraction. The baseline patient demographics are shown in Table 1. The indications for the extraction procedure are shown in Table 2. The mean patient age was 64 years old, 29% (7/24) of the patients were female and 29% (7/24) of the patients had prior sternotomy. Eleven high‐voltage leads underwent extraction, with nine of these being dual‐coil leads. The median lead dwell time was 16 years. The oldest lead in each extracted system ranged from 14 to 36 years. An excimer laser sheath was used in all extractions after lead pretreatment using a Philips GlideLight laser sheath, most often using a 14Fr sheath size. Rotational mechanical tools were used in four patients (seven leads total), in whom lead dwell time ranged from 10 to 25 years. As an illustration of the degree of calcification encountered, two of these patients required over an hour of tissue dissection to free the leads from the pocket alone.

**Table 1:** Baseline patient demographics.

Patient age	Gender	Prior sternotomy	Serum Cr	eGFR	LVEF
71	M	No	1.27	60	37
78	M	Yes	1.13	67	50
56	F	No	0.79	88	69
73	M	No	0.92	88	35
63	M	No	0.93	75	45
69	M	No	1.79	42	49
79	M	Yes	1.59	34	25
72	M	No	0.91	78	79
31	F	Yes	0.80	90	47
77	M	No	1.17	54	50
46	F	No	0.79	90	
65	M	No	1.20	70	60
59	M	Yes	1.01	86	60
71	M	No	0.86	72	55
61	M	No	1.24	66	57
56	M	Yes	0.89	90	29
48	F	Yes	2.66	21	55
52	F	No	1.12	59	55
63	F	Yes	1.13	55	55
72	M	No	1.09	72	40
76	F	No	0.78	90	45
82	M	No	1.49	57	55
26	M	No	1.41	70	50
86	M	No	1.82	36	55

Abbreviations: eGFR, Estimated glomerular filtration rate; LVEF, left ventricular ejection fraction.

**Table 2:** Extraction and device system details.

Patient age	Lead age (years)	Manufacturer	Chamber	Type	Fixation	Indication	Mechanical tool
71	22	Medtronic	RA	Bipolar	Passive	Pocket infection	Yes
	22	Medtronic	RV	Bipolar	Passive		Yes
78	35	Intermedics	RA	Unipolar	Active	Pocket infection	No
	35	Intermedics	RV	Unipolar	Active		No
56	36	Medtronic	RA	Bipolar	Passive	High threshold	No
	36	Medtronic	RV	Bipolar	Passive		No
	28	Medtronic	RV	Bipolar	Passive		No
73	30	Medtronic	RA	Bipolar	Passive	Device endocarditis	No
	30	Medtronic	RV	Bipolar	Passive		No
	4	Medtronic	RV ICD	Dual coil	Active		No
	4	Medtronic	CS	Quadripolar	Passive		No
63	20	Medtronic	RA	Bipolar	Active	SVC syndrome	No
	13	Medtronic	RV	Bipolar	Active		No
	13	Medtronic	RV ICD	Dual coil	Active		No
69	16	Abbott	RA	Bipolar	Active	Valve endocarditis	No
	16	Abbott	RV	Bipolar	Active		No
79	18	Medtronic	RA	Bipolar	Passive	Pocket infection	No
	18	Medtronic	RV	Bipolar	Active		No
	6	Medtronic	RV ICD	Single coil	Active		No
72	14	Abbott	RA	Bipolar	Active	Valve endocarditis	No
	14	Abbott	RV	Bipolar	Active		No
31†	7	Abbott	RA	Bipolar	Active	Severe TR	No
	15	Medtronic	RA	Bipolar	Active		No
77	28	Abbott	RA	Bipolar	Active	Pocket infection	No
	28	Abbott	RV	Bipolar	Passive		No
46*	25	Guidant	RV ICD	Dual coil	Passive	Patient request—LQT	Yes
	21	Guidant	RV ICD	Dual coil	Passive		Yes
65*	23	Guidant	RA	Bipolar	Passive	Lead fracture	No
	23	Guidant	RV	Bipolar	Passive		No
59	13	Abbott	RA	Bipolar	Active	Lead fracture	No
	13	Abbott	RV	Bipolar	Passive		No
71	10	Medtronic	RV	Bipolar	Active	Lead fracture	No
61	21	Abbott	RA	Bipolar	Active	Lead fracture	Yes
	21	Abbott	RV	Bipolar	Active		Yes
	4	Medtronic	RA	Bipolar	Active		No
	4	Medtronic	RV	Bipolar	Active		No
56	10	Medtronic	RV	Bipolar	Active	System upgrade	No
48	8	Medtronic	RA	Bipolar	Active	Severe TR, valve intervention	No
	8	Medtronic	RV	Bipolar	Active		No
52	10	Medtronic	RV	Bipolar	Active	Severe TR, valve intervention	Yes
63	16	Medtronic	RA	Bipolar	Active	Lead fracture	No
	16	Medtronic	RV	Bipolar	Active		No
	14	Medtronic	RV	Dual coil	Active		No
72	19	Guidant	RV	Dual coil	Active	Pocket infection	No
76	19	Guidant	RV	Dual coil	Active	Lead fracture	No
82	11	Medtronic	RV ICD	Dual coil	Active	Lead fracture	No
26	10	Medtronic	RV ICD	Dual coil	Active	Lead fracture	No
86	12	Medtronic	RA	Bipolar	Active	Pocket infection	No
	12	Medtronic	RV	Bipolar	Active		No

Abbreviations: ICD, implantable cardioverter defibrillator; RV, right ventricle.

* Indicates patients in whom we achieved clinical success, but not procedural success.

^†^ Indicates the patient in whom there was neither clinical nor procedural success.

Clinical success was achieved in 23 out of 24 patients, with procedural success in 21 of 24 patients. Eight cases were performed for lead fracture, with all lead components removed intact. Lead disruption during extraction was observed in four cases. In the first case, extraction of an abandoned 35‐year‐old unipolar atrial lead resulted in retention of the distal electrode. In the second case, extraction of an abandoned 20‐year‐old bipolar atrial lead resulted in retention of the distal electrode. Given the absence of systemic infection in these cases, the electrodes were left in vivo without an attempt to snare this portion. In the third case performed for CIED‐related endocarditis, a 30‐year‐old abandoned right ventricular lead unraveled during laser extraction despite the use of a locking stylet. A Needle's Eye Snare was advanced via right femoral venous access and was used to provide counter‐traction during laser application, with successful removal of all lead components. Finally, while extracting a 25‐year‐old dual coil passive fixation high voltage lead, the lead fractured two centimeters proximal to the distal electrode. The retained lead portion was removed with a Gooseneck Snare via a Medtronic Micra sheath with the lead, once again, fracturing and embolizing a tiny portion of lead coil to the right middle branch of the pulmonary artery. Given the elective nature of the extraction, this component was left without further attempt at retrieval.

In a single case, a right ventricular lead was abandoned in place. After freeing this lead from proximal vascular bindings, it was observed to be ensnared in the tricuspid valvular apparatus using intracardiac echocardiography. As the patient was scheduled for open aortic valve replacement the following day, the lead was clipped in the prepectoral pocket proximal to axillary venous access with surgical removal performed by direct visualization during the planned operation.

Minor complications occurred in two patients. One patient was noted to have superficial thrombophlebitis at a prior peripheral IV site 5 days after extraction. This was managed conservatively. A patient referred to our facility after a recently attempted extraction with retention of a large left ventricular lead segment was noted to have a small SVC dissection after successful system extraction at our facility. The dissection was in the SVC at the confluence of the right and left innominate veins and remained stable by venogram performed before and after biventricular system re‐implantation during the same procedure. No pericardial effusion was observed by serial echocardiograms, and no pleural effusion was noted on 2‐view chest radiography. No significant change in hemoglobin was observed on serial labs, and the patient was discharged in good condition the day after extraction.

Major complications occurred in a single patient. The patient became hypotensive following extraction requiring use of both excimer laser and mechanical sheaths. She required several sutures at the venotomy site to maintain hemostasis during the procedure, in addition careful cautery for diffuse oozing from the pectoral muscular plane. She was transferred to the intensive care unit requiring vasopressor support, which was weaned after transfusion of a single unit of packed red blood cells. She was diagnosed with a left anterior chest wall hematoma by CT imaging and was discharged home 5 days after the procedure.

Two patients underwent TLE due to high‐grade central venous stenosis. In the first patient with left innominate venous stenosis, initial venoplasty was performed with serial dilatation with a semi‐compliant balloon prior to IVL of the calcified lesion encasing the pacemaker leads with an 8 mm Shockwave balloon. Thereafter, successful laser‐assisted extraction was performed without complication. In the second patient, extraction was performed for SVC syndrome, which developed after implantation of bilateral device systems. Extraction was performed as described in methods above, with Shockwave IVL utilized for pretreatment within the right and left innominate veins. Following extraction, serial dilation of the right and left innominate veins using 10‐ and 12‐mm angioplasty balloons before placement of covered, self‐expanding stents in both innominate veins extending into the SVC. The stents were then postdilated with simultaneous inflation of 9 mm x 4 cm angioplasty balloons. A dual‐chamber conduction system pacing device was then placed without complication.

## 4 Discussion

While this is a small retrospective study, our real‐world outcomes using Shockwave IVL as adjuvant therapy is quite favorable. Although similar data has been previously presented, our patient population is comprised of CIED with leads having a longer average dwell time of 17 years (median 16 years) compared to a mean of 11.1 years in other published series [17]. As IVL was used at the operator's discretion, including cases selected after failed attempts with laser and or manual dissection with the VisiSheath, there is significant selection bias present in this dataset, with cases skewed to the most complex device systems. While safety and efficiency were not specifically studied as outcome, we experienced no adverse events and expect that IVL facilitated the removal of leads that may otherwise have required an open approach, including the extraction of 35‐year‐old unipolar Intermedics lead. A procedural success rate of 94% was achieved in the 49 leads removed, and the desired clinical endpoint was reached for all patients with continued follow‐up out to 22 months since the initial Shockwave IVL was performed.

Of note, the Shockwave balloon is indicated for use on calcified lesions in the arterial system. Its use in the venous system is not approved by the USFDA [19].

In our study, crossover from excimer laser sheaths as the first‐line extraction tool to a rotational mechanical tool was observed in approximately 12% of leads. Crossover from laser extraction tools to mechanical extraction tools has previously been reported as high as 19.5% in a prior direct head‐to‐head comparison in a single center using a patient population with a mean lead dwell time of 9.4 years without IVL pretreatment [12]. While this may represent site‐specific practice variability, it is suggestive of IVL pretreatment efficacy.

An area that requires additional evaluation is the potential impact that IVL may have on retained leads. In our study, all leads were removed in their entirety before replacing the device system. There were no cases in which IVL was used next to lead intended for ongoing service. It is possible that IVL could compromise long‐term lead integrity.

Finally, owing to the need for juxtaposition of lithotripsy catheter to the target, we did not attempt application of Shockwave within the cardiac chambers or coronary sinus. Whether it is feasible and efficacious to deliver IVL pretreatment to lead segments within the atrium or ventricles in sufficient proximity to the leads has yet to be seen based on our experience.

### 4.1 Study Limitations

This retrospective observational study represents a single‐center experience in a nonrandomized patient population of limited sample size. Our work describes the outcomes of IVL as an adjunct to conventional TLE in a high‐risk population with long lead dwell time, however, we did not compare predetermined endpoints of the IVL cohort to a standard TLE cohort. Further evaluation by a prospective randomized trial is warranted to further discern the difference in extraction times, fluoroscopy times, number and type of extractions tools, rate of clinical success and procedural complications with the addition of this technique. While our complication rate remained low in our limited series, we would not necessarily expect this finding to apply to the broader extraction population. Among other important considerations moving forward, whether IVL can be used with the intent of retaining hardware in the setting of system upgrade and whether IVL can be safely and effectively used in intracardiac chambers has yet to be answered.

The cost of the IVL device is significant and this needs to be considered in the analysis. It is modestly more that the cost of a laser sheath.

## 5 Conclusions

While additional work is warranted, our experience contributes to the growing body of evidence supporting Shockwave IVL as an adjunctive measure during extraction of high‐risk and high‐complexity systems with long lead dwell times.

## Disclosure

Dr. Crossley receives modest income for consulting and teaching for Medtronic. Dr. Richardson received teaching honoraria from Medtronic and serves as a consultant for Boston Scientific, Johnson & Johnson, and Abbott.
